# Iatrogenic Microperforation Linked to Hemorrhagic Pericardial Effusion Complicated by Cardiac Tamponade

**DOI:** 10.7759/cureus.9023

**Published:** 2020-07-06

**Authors:** Arun Philip

**Affiliations:** 1 Internal Medicine, Methodist Health System, Dallas, USA

**Keywords:** cardiac tamponade, pericardial effusion, pacemaker lead perforation

## Abstract

Pericardial effusions have a wide range of etiologies, including infection, inflammation, and malignancy. A complication of pericardial effusion is cardiac tamponade. In instances of cardiac tamponade, prompt echocardiography and stabilization are paramount in preventing mortality. Here, we report a case of iatrogenic microperforation of the right ventricle during a pacemaker lead adjustment causing a delayed pericardial effusion complicated by cardiac tamponade. Lead removal is recommended in cases of valvular endocarditis, pocket infection, thrombosis, or life-threatening dysrhythmias; however, there are no established guidelines in the setting of perforation. In this case, an emergent pericardiocentesis was performed due to cardiac tamponade, but lead extraction was not performed.

## Introduction

The pericardium is a sac surrounding the heart that contains a thin layer of fluid. A pericardial effusion occurs when there is an abnormally large amount of fluid in this layer. Pericardial effusions have a wide range of etiologies, including infection, inflammation, and malignancy [1]. Effusions can be acute or chronic, and the pericardium can expand with increases in fluid. They are challenging to identify as they can present asymptomatically to sudden cardiac death.

One of the most severe complications of a pericardial effusion is cardiac tamponade. Hemorrhagic pericardial effusions complicated by cardiac tamponade are typically caused by malignancy, percutaneous interventional procedures, post-pericardiotomy syndrome, and complications of myocardial infarctions [2]. Cardiac tamponade occurs when intrapericardial pressure increases to compress all cardiac chambers and impede cardiac filling. As tamponade progresses, intracardiac chamber volumes and diastolic compliance decreases resulting in decreased cardiac output. With further progression of cardiac tamponade, patients can suffer shock and death.

Perforations following pacemaker implantation can be defined as acute/early (<24 hours after implantation), subacute (up to one month), or chronic. Multiple case reports of early (<24 hours after implantation) iatrogenic right ventricle perforation after device implantation have been reported; however, this presentation is usually accompanied by chest pain and shortness of breath [3,4]. We present an uncommon cause of hemorrhagic pericardial effusion complicated by cardiac tamponade in which iatrogenic microperforation of the right ventricle occurred during a lead adjustment.

## Case presentation

**Figure 1:**
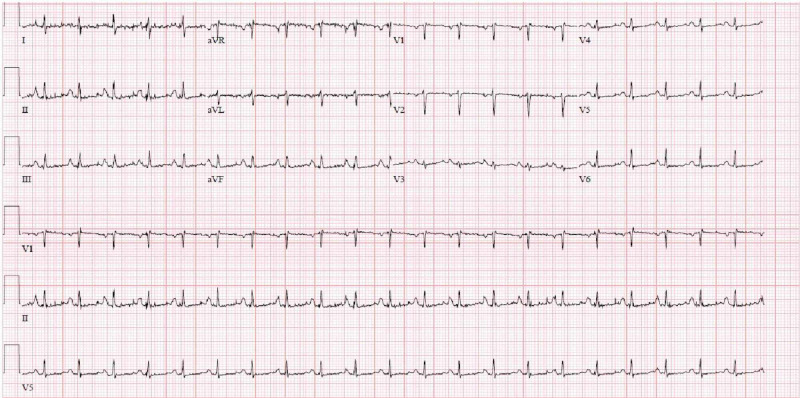
Electrocardiogram showing sinus tachycardia

A 77-year-old male presented to the emergency room with a two-week history of abdominal pain and dyspnea on exertion. The patient had a history of complete heart block with permanent pacemaker placement (two years prior to admission) and a recent diagnosis of nonvalvular atrial fibrillation on apixaban. Physical exam revealed that the patient was tachycardic. A complete blood count, electrolyte panel, and serum creatinine were unremarkable. The initial troponin value was elevated (0.033 mg/mL), and electrocardiogram showed sinus tachycardia (Figure 1). 

**Figure 2:**
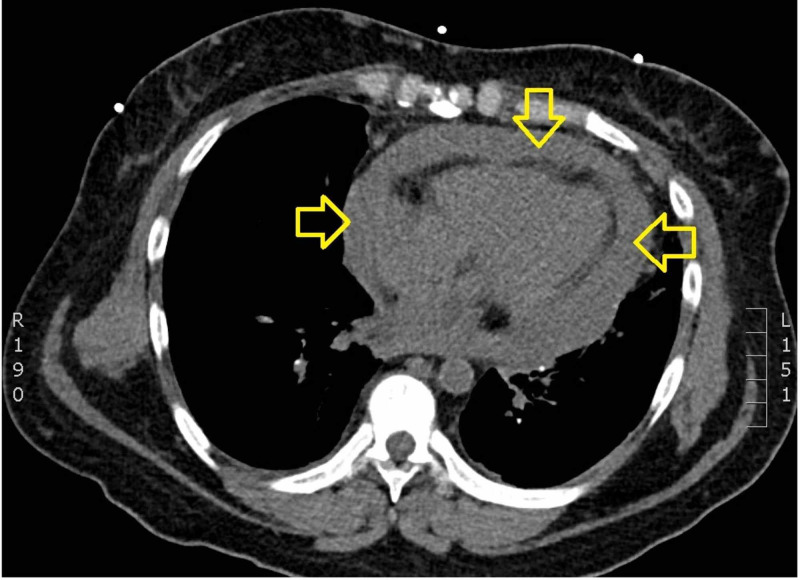
CT showing large pericardial effusion

CT of the chest revealed a pericardial effusion (Figure 2). 

**Figure 3:**
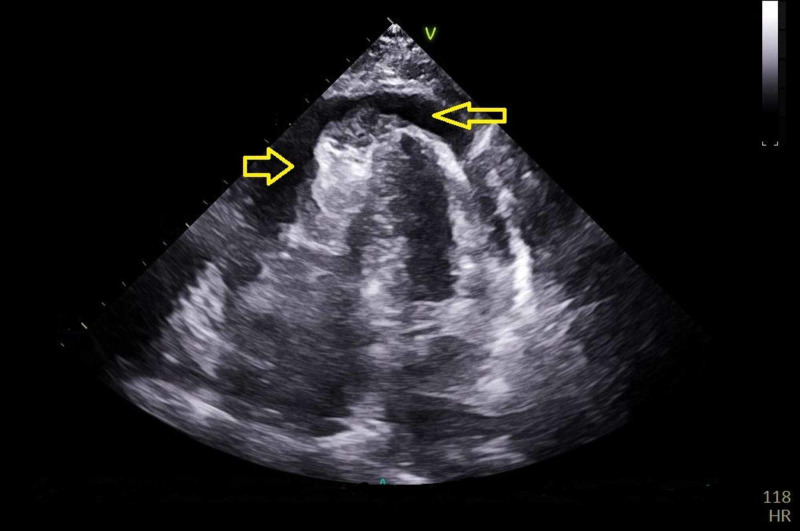
Transthoracic echocardiogram showing pericardial effusion

A transthoracic echocardiogram (TTE) showed circumferential pericardial effusion with findings consistent with cardiac tamponade (Figure 3).

**Figure 4:**
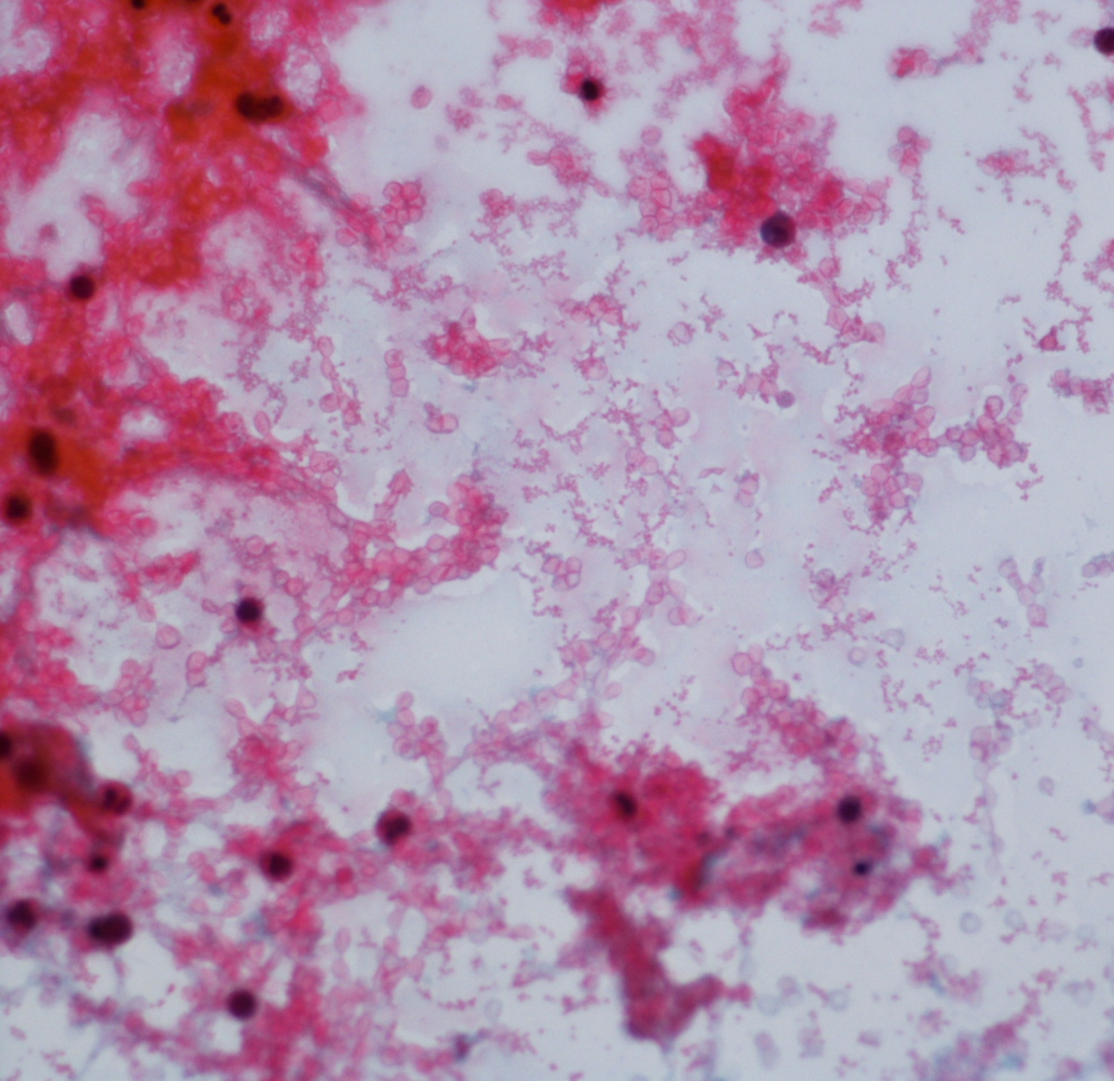
Fluid analysis of pericardial fluid shows abundant red blood cells with acute inflammatory cells with rare benign mesothelial cells. No malignant cells were identified.

Cardiology was consulted and emergent pericardiocentesis was performed, in which 560 mL of hemorrhagic fluid was drained from the effusion (Figure 4). 

A repeat TTE showed no pericardial effusion after drainage. Gram, fungal, and mycobacterial stains were negative, and bacterial, fungal, and mycobacterial cultures of the pericardial fluid showed no growth. Cytologic evaluation showed no evidence for malignancy and serum thyroid-stimulating hormone was normal. Together, these results ruled out hypothyroidism, malignancy, and infectious etiologies for the pericardial effusion. The patient’s records showed that his cardiologist had adjusted the patient’s pacemaker leads two weeks prior to admission. It was determined that the pericardial effusion was most likely iatrogenic due to microperforation of the right ventricle during pacemaker lead adjustment, with bleeding exacerbated by apixaban use. The patient’s abdominal pain resolved, and he was discharged home with outpatient cardiology follow-up. 

## Discussion

Myocardial perforation is a possible complication of permanent pacemaker implantation. Iatrogenic causations (e.g., transcatheter intervention and pacemaker insertion) have been implicated as causes of hemorrhagic pericardial effusions presenting with cardiac tamponade [1-3]. Other causes of pericardial effusions include malignancy (breast, lung, and lymphoma), complications from acute myocardial infarction, and uremia [3].

Right ventricular perforation is an uncommon cause of pericardial effusion, occurring in 0.1% to 0.8% of pacemaker placements [4-6]. Risk factors for ventricular perforation include the use of active fixation leads compared to passive fixation leads, low volume operators (less than 50 annual cases), female gender, steroid use, anticoagulant use, age greater than 75 years, body mass index (BMI) less than 25, chronic lung disease, and history of coronary intervention [7]. Symptoms of ventricular perforation can range from asymptomatic to life-threatening and include hiccups, chest pain, syncope, abdominal pain, and dyspnea [4].

Signs of right ventricle perforation include mammary hematoma, significantly decreased right ventricular pacing impedance, loss of ventricular capture (failure of generator stimulus to depolarize atrium or ventricle), and increased pacing threshold (observed on pacemaker interrogation) [4-6]. Chest x-rays can show migrated leads out of the heart silhouette as well as pleural or pericardial effusion, while TTE can show tips of leads in the pericardial space [6-8]. Fluoroscopy can be used to demonstrate abnormal motion or a hinge point of a lead trapped in the myocardium. CT can be helpful if other modalities are nondiagnostic; however, there can be difficulty in precisely locating the lead tip due to artifact [7,8].

In the current case, an emergent pericardiocentesis was performed due to tamponade; however, lead extraction was not deemed necessary during this admission. The necessity of lead extraction, and the optimal means of extraction, whether percutaneous or surgical, remains a matter of controversy [4]. Lead extraction is necessary in patients with life-threatening dysrhythmias, when the lead interferes with the operation of implanted cardiac devices, and if the lead interferes with treatment of a malignancy [9,10]. Many cases can be managed by lead repositioning; however, increased length of time since implantation is associated with an increased risk of fibrotic adhesions. Recent guidelines suggest if the lead tip is inside the mediastinum and there is no active bleeding, an additional lead can be placed without performing lead extraction. However, the potential for further migration needs to be evaluated. If uncontrolled bleeding occurs or if the lead migrates outside the pericardium, then lead extraction must be performed [9]. Cardiotomy with surgical removal is typically reserved for cases in which transcutaneous approaches have failed, for hemodynamically unstable patients, or for patients with respiratory compromise [8-10]. 

## Conclusions

Cardiac perforation is an extremely rare etiology of pericardial effusion; however, it is associated with significant morbidity and mortality. It should be suspected in patients presenting with a history of recent cardiac intervention and hemorrhagic pericardial effusion.
